# The simultaneous perception of self- and non-self-danger signals potentiates plant innate immunity responses

**DOI:** 10.1007/s00425-022-03918-y

**Published:** 2022-06-13

**Authors:** Victoria Pastor, Raquel Cervero, Jordi Gamir

**Affiliations:** Metabolic Integration and Cell Signaling Group, Departamento de Biología, Bioquímica y Ciencias Naturales, University Jaume I of Castellón, 12071 Castelló de la Plana, Spain

**Keywords:** cAMP, Cellobiose, DAMPs, Flagellin, MAPK, PAMPs, *Pseudomonas syringae*, PTI, ROS

## Abstract

The simultaneous perception of endogenous and exogenous danger signals potentiates PAMP-triggered immunity in tomato and other downstream defence responses depending on the origin of the signal.

**Abstract** Plant cells perceive a pathogen invasion by recognising endogenous or exogenous extracellular signals such as Damage-Associated Molecular Patterns (DAMPs) or Pathogen-Associated Molecular Patterns (PAMPs). In particular, DAMPs are intracellular molecules or cell wall fragments passive or actively released to the apoplast, whose extracellular recognition by intact cells triggers specific immune signalling, the so-called DAMP-triggered immunity. The extracellular recognition of DAMPs and PAMPs leads to a very similar intracellular signalling, and this similarity has generated a biological need to know why plants perceive molecules with such different origins and with overlapped innate immunity responses. Here, we report that the simultaneous perception of DAMPs and a PAMP strengthens early and late plant defence responses. To this aim, we studied classical PTI responses such as the generation of ROS and MAPK phosphorylation, but we also monitored the biosynthesis of phytocytokines and performed a non-targeted metabolomic analysis. We demonstrate that co-application of the bacterial peptide flagellin with the DAMPs cyclic AMP or cellobiose amplifies PAMP-triggered immunity responses. Both co-applications enhanced the synthesis of phytocytokines, but only simultaneous treatments with cAMP strengthened the flagellin-dependent metabolomic responses. In addition, cAMP and cellobiose treatments induced resistance against the hemibiotrophic bacteria *Pseudomonas syringae* pv. *tomato* DC3000. Overall, these results indicate that the complex mixture of DAMPs and PAMPs carries specific information that potentiates plant defence responses. However, downstream responses seem more specific depending on the composition of the mixture.

**Supplementary Information:**

The online version contains supplementary material available at 10.1007/s00425-022-03918-y.

## Introduction

Plants activate the innate immune system after the recognition of molecular patterns. The self-damage perception occurs after sensing Damage-Associated Molecular Patterns (DAMPs) that induces DAMP-Triggered Immunity (DTI) (Hou et al. 2019). Alternatively, the molecular recognition of a pathogen occurs after the perception of conserved pathogen-specific molecules, the so-called Pathogen-Associated Molecular Patterns (PAMPs), inducing PAMP-Triggered Immunity (PTI) in plants (Boller and He 2009; Zhang and Zhou 2010; Ranf 2017; Zipfel and Oldroyd 2017; Yu et al. 2021). In both cases, the recognition of self- and non-self-molecular signatures entails the activation of plant innate immunity, enhancing plant tolerance against a broad range of pathogens and insects (Benedetti et al. 2015; Duran-Flores and Heil 2016; Bacete et al. 2018; Gamir et al. 2021). Interestingly, PTI- and DTI-associated responses have been so far studied separately, but the benefits of the simultaneous recognition have not been well described yet.

Damage-associated molecular patterns (DAMPs) are host-derived molecules produced upon herbivory or pathogen infection. These molecules are released to the apoplast after a pathogen or insect attack, and their perception in adjacent intact cells triggers an alarm state in the plant (Heil and Land 2014; De Lorenzo et al. 2018). DAMPs are classified in primary or secondary depending on their origin. Primary DAMPs are intracellular molecules, such as nucleotides, extracellular ATP (eATP), extracellular DNA (eDNA), cyclic AMP (cAMP), NAD^+^ or cell wall fragments, cellobiose, oligogalacturonides and β-glucans, that are passively leaked to the extracellular cell space after cell disruption (Gust et al. 2017). On the other hand, secondary DAMPs, or phytocytokines, are inducible peptides synthesized or processed after cell damage that are actively released to the apoplast. For instance, SYSTEMIN (SYS) is a hormonal tomato peptide that accumulates after a predator attack inducing proteinase inhibitor activity similar to wounding or methyl jasmonate (Bishop et al. 1984; Ryan and Pearce 2003; Pastor et al. 2018). The perception of primary or secondary DAMPs activates conserved DTI responses such as Reactive Oxygen Species (ROS) burst, MAPK phosphorylation and Ca^2+^ signalling (Orozco-Cardenas and Ryan 1999; Barbero et al. 2016; Bacete et al. 2017; Shinya et al. 2018; Tripathi et al. 2018; Pastor-Fernández et al. 2020). However, DAMPs sensing can also elicit specific defence responses, according to their origin.

When pathogens cross the plant cell wall to access the cytoplasm, many different endogenous molecules are liberated to the apoplast. Cellobiose is a degradation product of cellulose, one of the major components of the cell wall. The extracellular perception of cellobiose activates DTI responses in Arabidopsis and, simultaneous treatments of cellobiose and a PAMP increases Ca^2+^ influx compared to individual treatments (Souza et al. 2017). Alternatively, the perception of extracellular nucleotides also triggers an alarm state in intact cells. However, there is scarce information about DTI responses triggered by nucleotides, and only the extracellular receptor for ATP has been discovered (Choi et al. 2014). The role of cAMP as an intracellular signal in plant defence has been suggested in very few studies so far. Higher levels of cAMP correlate with the activation of defence-related responses such as an increase in salicylic acid levels, production of phytoalexins, and stimulates pathogen-induced ROS (Zhao et al. 2004; Blanco et al. 2020). In addition, exogenous cAMP application induces defence response gene expression, all of which conserved defence responses similar to PAMPs-triggered immunity (Ma et al. 2009).

After damage perception, plants enter an alarm state boosting the first layer of defences and inducing local and systemic resistance against detrimental enemies. For example, nucleotide applications such as eDNA and eATP induce plant resistance against virus and bacteria in different plant species (Wen et al. 2009; Tripathi et al. 2016; Duran-Flores and Heil 2018; Rassizadeh et al. 2021). Additionally, the application of cell wall-derived oligogalacturonides induces not only local resistance in Arabidopsis but also systemic resistance in tomato plants against the necrotrophic pathogen *B. cinerea* (Aziz et al. 2004; Ferrari et al. 2007; Gamir et al. 2021). A recent study demonstrated that *Arabidopsis response regulator 6* (*arr6-3*) mutant lines are more susceptible against *Plectosphaerella cucumerina* than control plants (Bacete et al. 2020; Rebaque et al. 2021). This mutation triggered an altered biochemical composition of the cell wall, indicating that cell-wall signalling is also crucial for plant defence. DAMPs are considered future plant vaccines to protect crops against pests and diseases (Quintana-Rodriguez et al. 2018). However, we still do not know whether DAMPs provide broad-range resistance, pathogen-specific resistance or whether the resistance provided by DAMPs depends on the origin of the signal. One plausible hypothesis is that DAMPs recognition in plants may not trigger specific responses against an invader, since it does not provide any information about the pathogen. However, the simultaneous perception of DAMPs and PAMPs may provide specific information about the damage and the enemy and this combination will trigger defence specific responses (Duran-Flores and Heil 2016).

PAMPs are conserved structures of pathogens perceived by pattern recognition receptors (PRR) which activate PTI responses (Zipfel and Oldroyd 2017). The perception of PAMPs constitutes the first layer of the plant innate immunity that usually triggers a downstream signalling cascade. For example, the recognition of flagellin, the major component of the bacteria flagella, triggers ROS production, MAPK phosphorylation in *Arabidopsis thaliana.* Additionally, inoculation with the non-pathogenic bacterium *Pseudomonas fluorescens* and type III secretion mutant DC3000*ΔhrcQ*_*b*_*RSTU* induces the expression levels of the transcription factors Pto Interacting protein 5 (Pti5) and GRAS2 (Nguyen et al. 2010; Rosli et al. 2013; Sun et al. 2013). To date, two flagellin receptors have been described. The flagellin receptor FLAGELLIN-SENSING 2 (FLS2) is a leucine-rich repeats-receptor kinase (LRR-RK), which, after binding to its ligand, flg22, interacts with BRI1-ASSOCIATED RECEPTOR KINASE 1 (BAK1), another LRR-RK (Chinchilla et al. 2006; Sun et al. 2013). Recently, a new flagellin receptor has been identified in tomato, FLAGELLIN-SENSING 3 (FLS3), which binds to a distinct ligand, flgII-28 (Fliegmann and Felix 2016; Hind et al. 2016). Like cellobiose and other DAMPs, flagellin triggers ROS burst, MAPK phosphorylation and callose deposition after its recognition (Zhang et al. 2020). A few years ago, Duran-Flores and Heil (2016) proposed four non-exclusive hypotheses of why plants can recognize PAMPs and DAMPs to trigger similar/conserved downstream responses, but there is very little scientific evidence to confirm any of them. It has recently been shown that Arabidopsis roots increase the number of extracellular membrane receptors in intact adjacent cells after the perception of a nematode Microbe-Associated Molecular Pattern (MAMP) (Zhou et al. 2020). This result is the first molecular evidence showing the advantage to recognize both signals. However, it is necessary to have more molecular and metabolic information to understand the complete process behind DAMPs and PAMPs recognition.

In the present manuscript, we aim at demonstrating that simultaneous perception of DAMPs and PAMPs amplifies downstream PTI responses in tomato. It has been described that DAMPs and PAMPs activate defence signalling when recognised independently, and many hypotheses have been proposed to understand why plants have evolved the capacity to sense both signals. However, very little experimental evidence is available to corroborate any proposed hypothesis (Felix et al. 1993; Cabrera et al. 2010; Duran-Flores and Heil 2016; Azevedo-Souza et al. 2017). To this aim, we analysed classical PTI responses in tomato, such as ROS accumulation, MAPK phosphorylation, and the expression of PTI reporter genes in tomato treated with the bacterial PAMP flagellin (flg22) and two different DAMPs, the cell wall derivative cellobiose and the nucleotide cAMP. Additionally, we quantified the levels of secondary DAMPS and performed a non-targeted metabolomic analysis. Finally, we performed a disease resistance bioassay against the bacterial pathogen *Pseudomonas syringae* pv*. tomato* DC3000 (*Pst*) in plants treated with cellobiose or cAMP. Our results show that the co-application of both signals boosts PTI responses compared to individual DAMP or PAMP application, indicating that the simultaneous perception of both signals may enhance plant protection against bacterial pathogens. Overall, we demonstrate that, biologically, the plants have evolved to sense self-damage recognition and external traits to potentiate innate immune responses, helping them cope with detrimental microbes.

## Materials and methods

### Plant materials and treatments

Tomato seeds (*Solanum lycopersicum* cv *Castelmart*) were surface sterilized in 4% sodium hypochlorite, rinsed thoroughly with sterile water and grown in 100 mL plastic pots with sterile vermiculite. The seeds were watered with distilled water during the first 2 weeks and then irrigated with Hoagland solution (Hoagland and Arnon 1950). Tomato plants were grown in a growth chamber with a 16/8 h light cycle at 25/19ºC temperature and 60% relative humidity. Flagellin peptide (Q-R-L-S-T-G-S-R-I-N-S-A-K-N–N-A-A-G-L-Q-I-A) (GeneScript) stock was prepared at 100 mM, cellobiose stock at 100 mM and cAMP stock at 6 mM. All the stocks were prepared in distilled water, aliquoted and stored at -20ºC and diluted in distilled water at the working concentration. Unless mentioned otherwise, we applied flg22 at 100 nM, cAMP at 25 µM and cellobiose at 100 µM.

### ROS determination

Luminol-based assays for the detection of ROS was performed according to Torres et al. (2013). We prepared leaf disks of tomato plants with a small size cork borer (3.5 mm diameter), placed them in a Petri dish filled with water, covered with a transparent lid and incubated overnight in the growth chamber. The following day, we transferred the disks in a 96-well plate and kept them in the dark. Meantime, we prepared 1 mL of an assay solution with water, 100 µM luminol, 10 µg/mL horseradish peroxidase, flagellin at 100 nM and the rest of elicitors as described in the figures. We added 100 µL of the assay solution to each well with a multichannel pipette in a low light environment and rapidly measured with a Luminoskan^™^ Microplate Luminometer (Thermo Fisher) for 2 h. Cumulative RLU represents total ROS production over the 80 min time-course as described in Steinbrenner et al. (2020). Eight biological replicates per sample were taken for each individual experiment and all the measurements were repeated at least three time with comparable results.

### MAPK assays

Four-week-old tomato plants were grown under the conditions previously described. The day before the treatments, we detached tomato leaflets from the third and fourth true leaves and kept them O/N in water. The next day, we sprayed flg22 at 100 nM, cellobiose at 100 µM, cAMP at 25 µM and the mixture solutions of cellobiose + flg22 and cAMP + flg22 at the same concentrations to the leaflets and harvested them in liquid nitrogen at the indicated timepoints. The frozen seedlings were ground in liquid nitrogen and homogenized in 100 µL of extraction buffer at 1:3 ratio (w/v) in the extraction buffer: 20 mM Tris–HCl, pH 7.5, 150 mM NaCl, 1 × Triton X-100, 10 mM DTT, 1 mM PMSF, 5 µL/mL proteinase/phosphatase inhibitor cocktail (Sigma-Aldrich). After centrifugation at 16,000 g for 30 min at 4 °C, we diluted protein supernatants with loading buffer and subjected them to SDS-PAGE and electroblotting. The immunoblots were saturated with 5% non-fat milk prepared in TBS-Tween 0.1%, and the analysis was performed using anti-phospho-p44/42 MAPK (1: 3,000; Cell Signaling Technology) and anti-AtMPK6 (1: 5,000; Sigma-Aldrich) as primary antibodies and peroxidase-conjugated goat anti-rabbit IgG (1: 15,000; catalog no. A 6154; Sigma-Aldrich) as a secondary antibody.

### Metabolomic analysis

The third and fourth true leaves of 4-week-old tomato plants were sprayed with PAMPs and DAMPs and harvested in liquid nitrogen 48 h after the treatments. The samples were homogenized in liquid nitrogen and extracted in H_2_O:MeOH:HCOOH (70:30:0.01, by vol.). Three hundred milligrams of fresh tissue were incubated with 1 mL of the extraction solution for 45 min and subsequently centrifuged for 20 min at 12,000 g, 4 ºC. The clear supernatants were collected and stored at – 20 ºC until the analysis. The untargeted metabolomics was performed using a Kinetex 2.6 µm EVO C18 UPLC column (Phenomenex Inc.) in the ACQUITY UPLC I-Class System and a SYNAPT G2-S high definition mass Spectrometer MS/MS detector (Waters^®^). After data acquisition, the files were transformed to CDF and processed with R and MetaboAnalyst.

### Gene expression analysis

The third and fourth true leaves of 4-week-old tomato plants were sprayed with DAMPs, flg22 and a mixture of DAMPs + PAMPs and harvested in liquid nitrogen 1, 3 and 6 h after the treatments. The samples were homogenized in liquid nitrogen, and the RNA was extracted following Pastor et al. (2020). Briefly, 100 µg of fresh tissue was incubated in 1 mL of TRIZOL and centrifuged for 5 min at room temperature. The supernatant was mixed with chloroform and centrifuged again. The supernatant was mixed with isopropanol (1:1, v/v) and incubated for 5 min at room temperature for RNA precipitation. The RNA was pelleted at 12,000 g for 10 min at 4 ºC, cleaned with EtOH, let dry at room temperature for 20 min and dissolved in 50 µL of nuclease-free water. The synthesis of cDNA was performed using a High Capacity cDNA Reverse Transcription Kit (Applied Biosystems). Quantitative Real-Time PCR (qPCR) was performed with a Maxima SYBR Green/ROX qPCR Master Mix (2X) (Thermo Fisher Scientific), using a StepOne instrument (Applied Biosystems). Relative quantification of specific mRNA levels was performed using the comparative 2–Δ(Δ*Ct*) method (Livak and Schmittgen 2001). We measured the expression of three different housekeeping genes, actin (Solyc03g078400), elongation factor 1-α (Solyc06g005060) and β-tubulin (Solyc04g081490) and, to find the optimal normalization gene among these three, we used the NormFinder software (https://moma.dk/normfinder-software). NormFinder is an algorithm for identifying the optimal normalization gene among a set of candidates. According to the results, expression values were normalized using the housekeeping gene elongation factor 1-α (EF-1α).

### Pseudomonas infection

Tomato plants were infected with *Pst*, according to Scalschi et al. (2020). Briefly, the third and fourth fully developed leaves from tomato plants were exogenously sprayed with MgSO_4_ as a control, cAMP, cellobiose and a mixture solution of cAMP and cellobiose 24 h before the infection. *Pst* was extracted from glycerol stock in King’sB solid media enriched with rifampicine 50 mg/mL and cycloheximide 100 mg/mL and incubated at 28° for 24 h. Then, we brought the bacteria to liquid King’sB media supplemented with rifampicine (50 mg/mL) and incubated for 3-4 h at 180 rpm. After the incubation, we estimated the bacterial colonies by measuring the optical density at 600 nm and prepared a bacterial solution in MgSO_4_ of 1 × 10^5^ cfu. The infection in tomato leaves was performed by submerging the third and fourth true tomato leaves into the bacterial solution for 3 s. The incidence of the infection was quantified at timepoints 0 h and 4 days after the infection. We counted the number of cfu in solid King’sB media (supplemented with rifampicine 50 mg/mL and cycloheximide 100 mg/mL) after the maceration of infected leaves at the concentration of 50 mg/mL of MgSO_4_. Serial dilutions of bacterial suspension (1:10, 1:100 and 1:1000) were plated in media plates and incubated at 28 ºC for 24 h.

### Peptide quantification

The protocol for the extraction and quantification of systemin and HypSYS1 was performed as indicated in Pastor et al. (2018) with slight modifications. Briefly, we homogenized 300 mg of fresh tomato leaves, stored at − 80 °C, and added 1.5 mL of phenol/TRIS saturated at pH = 8 (ACROS Organic, ref. 327,125,000) and 50 µL at 0.1 µM of the SYS heavy isotope [13C5, 15 N]-SYS (SYS*) as an internal standard (Genscript). After 40 min of incubation on ice, we centrifuged the samples for 15 min, 12,000 g at 4 ºC and filtered the supernatant with hydrophilic PVDF filter with a 25-mm diameter and a pore size of 0.45 µm (FILTER-LAB, La Rioja, Spain). Then, we added six volumes of pure cold acetone (Scharlau, AC0312, Pharm pur^®^) to each sample and kept them overnight at − 20 °C. The following day, we centrifuged the tubes for 20 min, 1956 g at 4 ºC, and the precipitates were rinsed twice with 2 mL of cold acetone. The liquid phase was discarded, and the pellet was dried. The final residue was re-suspended in 500 µL of a solution of H_2_O (HCOOH 0.1%): acetonitrile (9:1, v/v) and injected into the TQS-MS/MS instrument. The column, mobile phases and chromatographic conditions were identical to Pastor et al. (2018).

### Statistical analysis

Statgraphics was used for statistical analysis. Data were expressed as the mean ± standard error (SE) for the three replicates in each treatment. Statistical tests were performed using Student's *t* test or one-way analysis of variance (ANOVA), followed by Fisher’s least significant difference (LSD) at 99.5%. A probability (*P*) value of < 0.05 was considered significant.

## Results

### DAMPs application amplifies flagellin-induced ROS responses

**Fig. 1:**
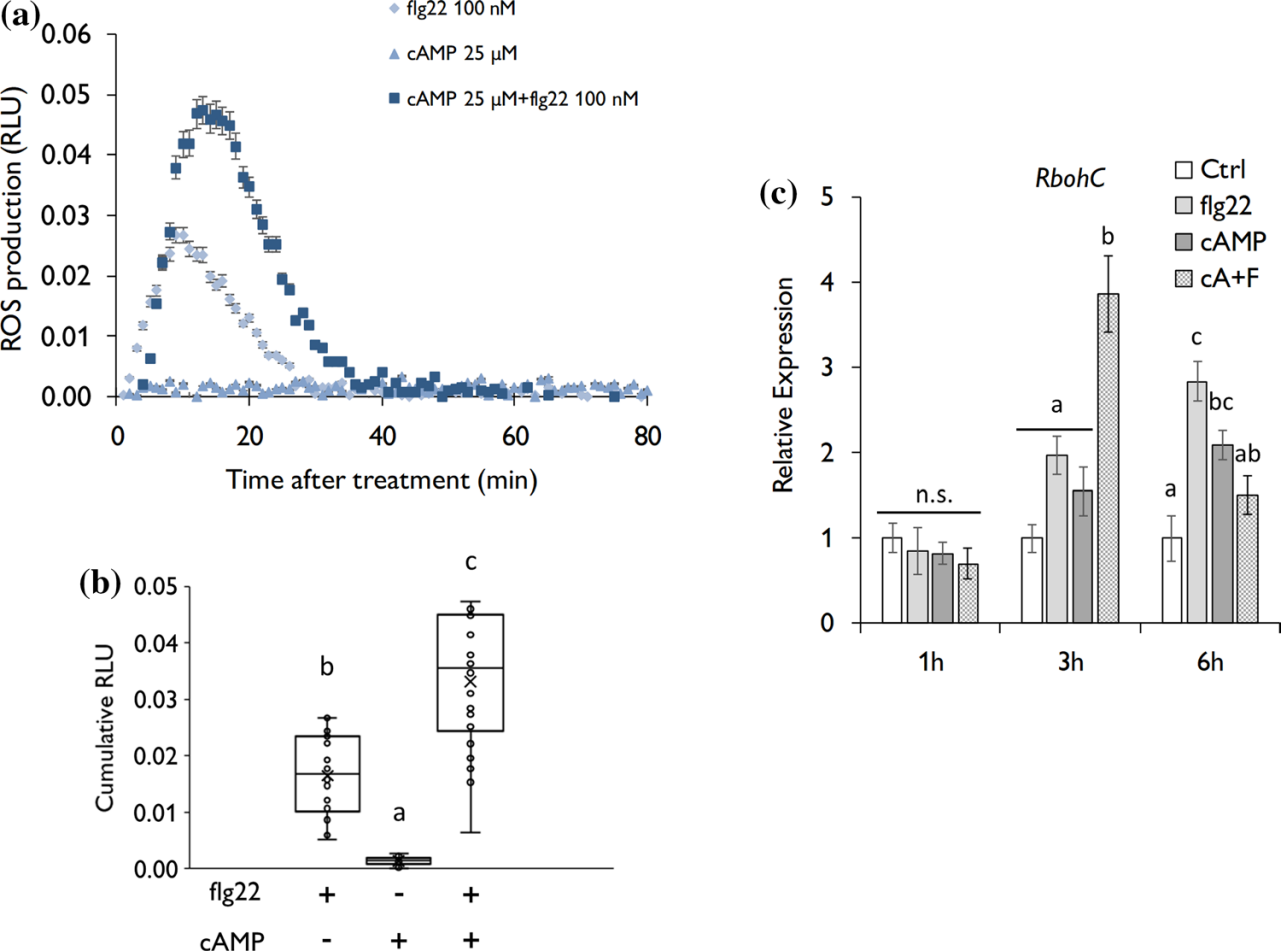
cAMP and flg22 co-application boosts ROS production. **a, b** ROS production in leaf disks determined from 4-week-old tomato elicited by the 22 amino acid flagellin peptide (flg22), cAMP and simultaneous application of flg22 and cAMP (*n* = 16 leaf disks). **a** Points represent means ± SE. **b** Integrated ROS production over 30 min. Line represents mean, error bars represent SD. **c** *RbohC* gene expression data quantified by RT-q-PCR 1,3 and 6 h after treatment. Water (Ctrl), flagellin peptide (flg22), and cAMP treatments were applied individually. A mixture of cAMP and flg22 (cA + F) was prepared for simultaneous application. Error bars represents SD; letters indicate statistical differences within the treatments (ANOVA, LSD *P* < 0.01; *n* = 3)

**Fig. 2:**
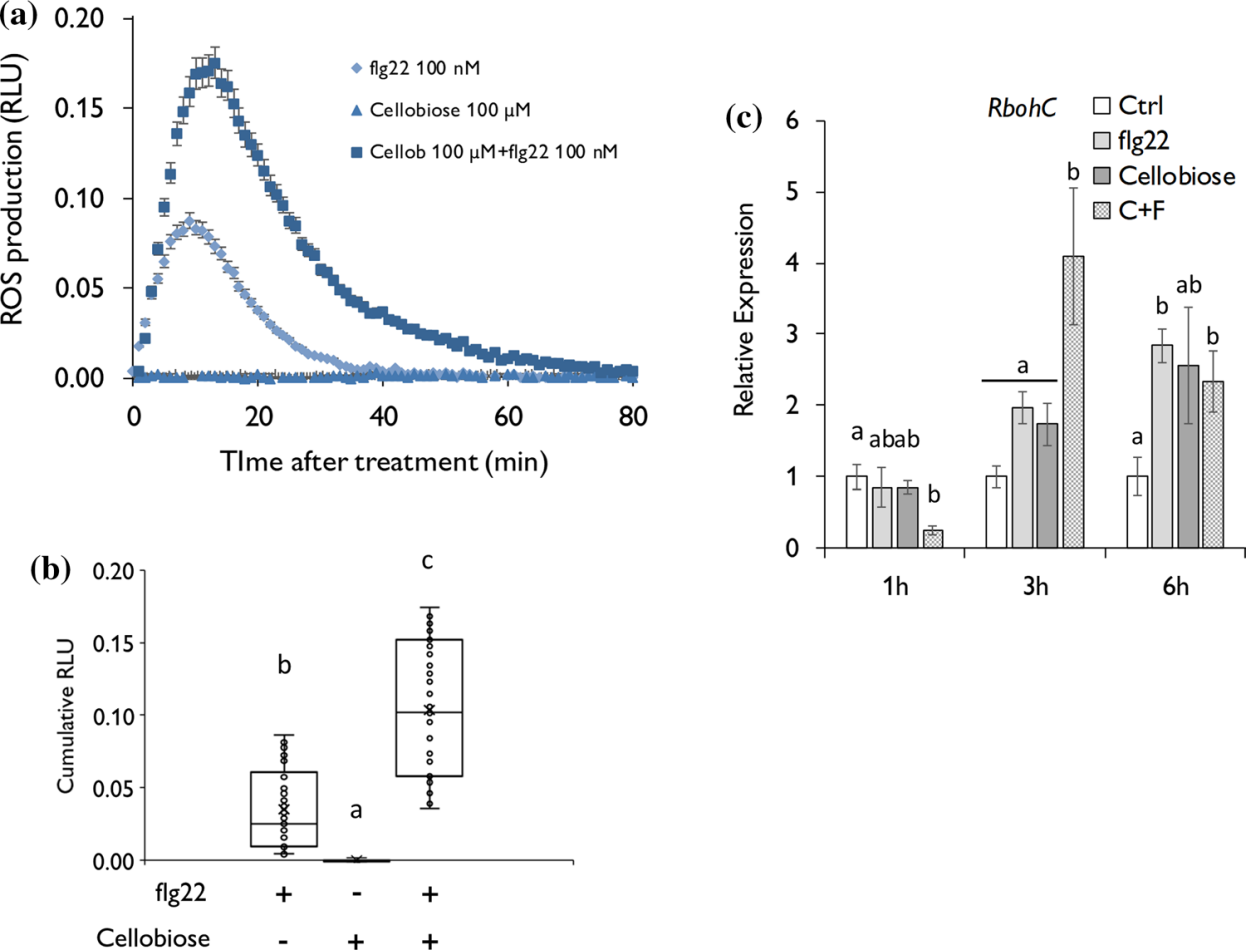
Cellobiose and flg22 co-application boosts ROS production. **a, b** ROS production in leaf disks determined from 4-week-old tomato elicited by the 22 amino acid flagellin peptide (flg22), cellobiose and simultaneous application of flg22 and cellobiose (*n* = 16 leaf disks). **a** Points represent mean values ± SE. **b** Integrated ROS production over 30 min. Line represents mean; error bars represent SD. **c** *RbohC* gene expression data quantified by RT-q-PCR 1,3 and 6 h after treatment. Water (Ctrl), flagellin peptide (flg22), and cellobiose treatments were applied individually. A mixture of cellobiose and flg22 (C + F) was prepare for simultaneous application. Error bars represent SD; letters indicate statistical differences within the treatments (ANOVA, LSD *P* < 0.01; *n* = 3)

To investigate whether the perception of DAMPs amplifies PTI responses, we monitored the generation of ROS after simultaneous application of the bacterial peptide flg22 as a PAMP, and we used two DAMPs from different origins; the nucleotide cAMP and the cellulose-derived oligomer cellobiose. Our results showed that the individual application of cAMP did not trigger extracellular H_2_O_2,_ but the simultaneous application of 100 nM of flg22 and 25 µM of cAMP did amplify flg22-dependent ROS production in tomato (Fig. 1a, b). Lower cAMP concentrations were also tested being only significant from 12.5 µM, and, interestingly, low cAMP concentrations reduced flg22-induced ROS (Fig. S1). The highest concentrations of cAMP tested (25 µM) do not provide higher ROS levels, being similar to the previous concentration of cAMP (12 µM). According to this observation, we monitored the expression of several *Respiratory Burst Oxidase Homologs* (*Rboh’s*) that respond to pathogen attack at 1,3 and 6 h post-treatment (hpt) (Li et al. 2015). *RbohA* and *RbohB* expression was upregulated 1 h after flg22 and cAMP treatments, but the mixture solution of flg22 and cAMP did not enhance the expression of both genes compared to individual treatments (Fig. S2). Instead, plants treated with flg22 and cAMP simultaneously showed enhanced expression of *RbohC* than plants with individual treatments at 3 hpt (Fig. 1c). In the same line, we observed a comparable response after the co-application of flg22 and cellobiose. We treated tomato leaves with a concentration of 100 nM of flg22 and 100 µM of cellobiose based on previous studies (Azevedo Souza et al. 2017). The simultaneous application of both compounds triggered higher production of extracellular ROS than flg22 alone (Fig. 2a, b), while cellobiose could not generate any response (Fig. 2a, b). The expression of *RbohC* was potentiated after DAMP and PAMP co-application 3 hpt compared to the individual application (Fig. 2c). Together, these data indicate that a simultaneous perception of DAMPs and PAMPs amplifies ROS generation in tomato.

### Co-application of DAMPs and PAMPs boosts PTI responses compared to individual treatments

**Fig. 3:**
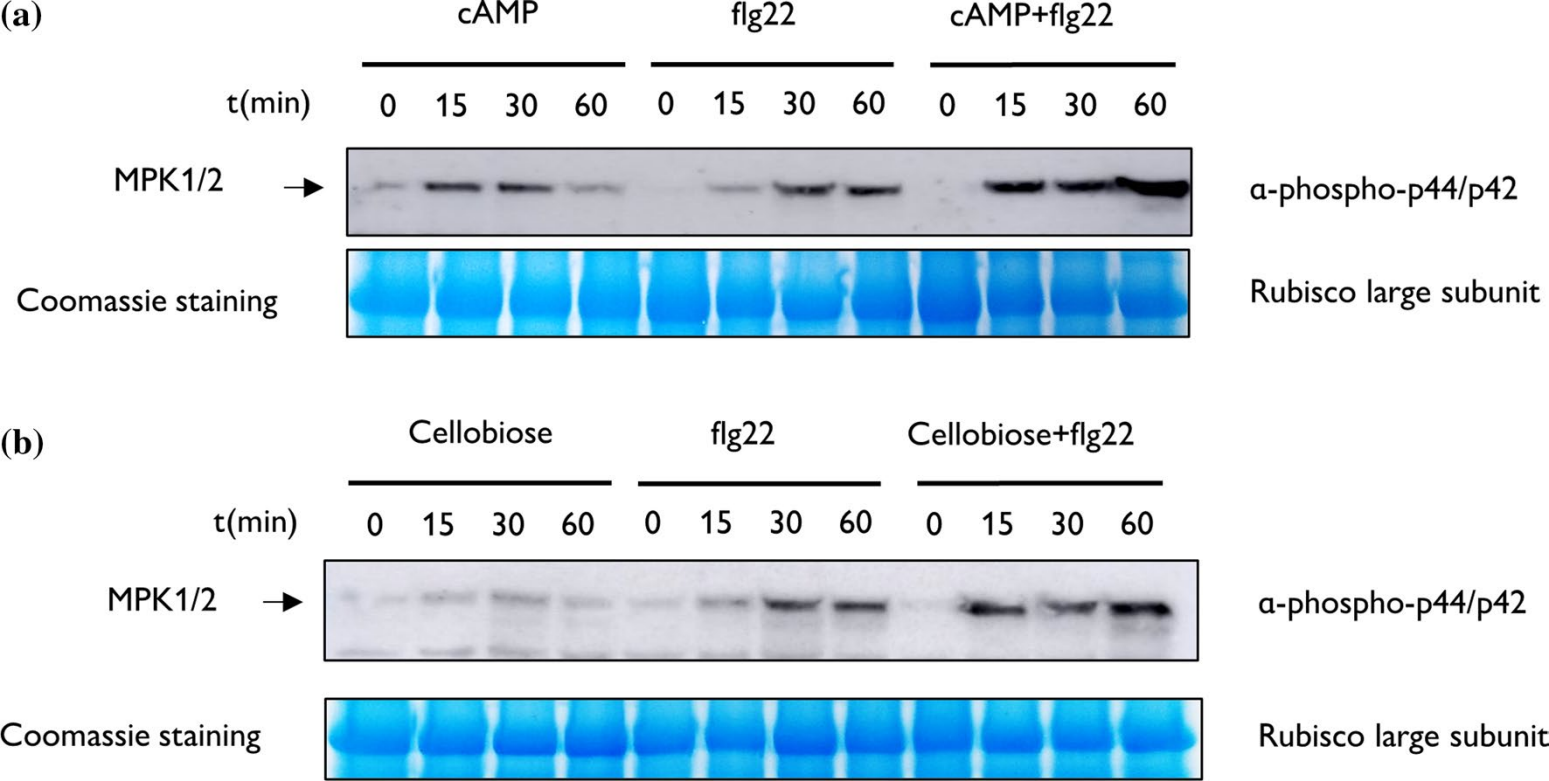
DAMP and PAMP simultaneous application amplifies the phosphorylation pattern of MPK1/2. Western blot using α-p44/p42 recognizing phosphorylated MPK1/2 in tomato plants treated with **a** cAMP, the 22 amino acid flagellin peptide (flg22) and a mixture solution of flg22 and cAMP (cAMP + flg22) and **b** cellobiose, the 22 amino acid flagellin peptide (flg22) and a mixture solution of cellobiose and flg22 (Cellobiose + flg22). Tomato leaves were harvested 0, 15, 30 and 60 min after treatment, and Coomassie staining in polyacrylamide protein gels was used as a loading control

To corroborate whether the simultaneous perception of self- and non-self-danger signals may potentiate PTI responses, we studied the MAPK phosphorylation pattern during the first hour after the treatments. MAPK regulates PTI responses that are often activated by phosphorylation (Rasmussen et al. 2012). Hence, we sprayed the third and fourth true leaves of tomato plants with an individual solution of DAMPs, cellobiose or cAMP, a solution of flg22 as a PAMP and a mixture solution of cAMP + flg22 or cellobiose + flg22. The western-blot analysis using α-p44/p42 revealed that cAMP and flg22 alone induce the phosphorylation of MAPK proteins 15 min after their application (Fig. 3a). However, simultaneous application of both elicitors showed higher phosphorylation after 15 min and increasing levels of phosphorylation after 60 min, in contrast to individual treatments where phosphorylation remained similar or even decreased (Fig. 3a). These effects were also visible for cellobiose treatments. Although tomato leaves treated with cellobiose did not induce MPK1/2 phosphorylation as flg22 did, simultaneous application of cellobiose and flg22 potentiated MAPK phosphorylation already after 15 min (Fig. 3b). Similar to cAMP, a higher accumulation of phosphorylated MPK1/2 was also visible 60 min after the simultaneous application of cellobiose and flg22 compared to cellobiose or flg22 treatments alone.

**Fig. 4:**
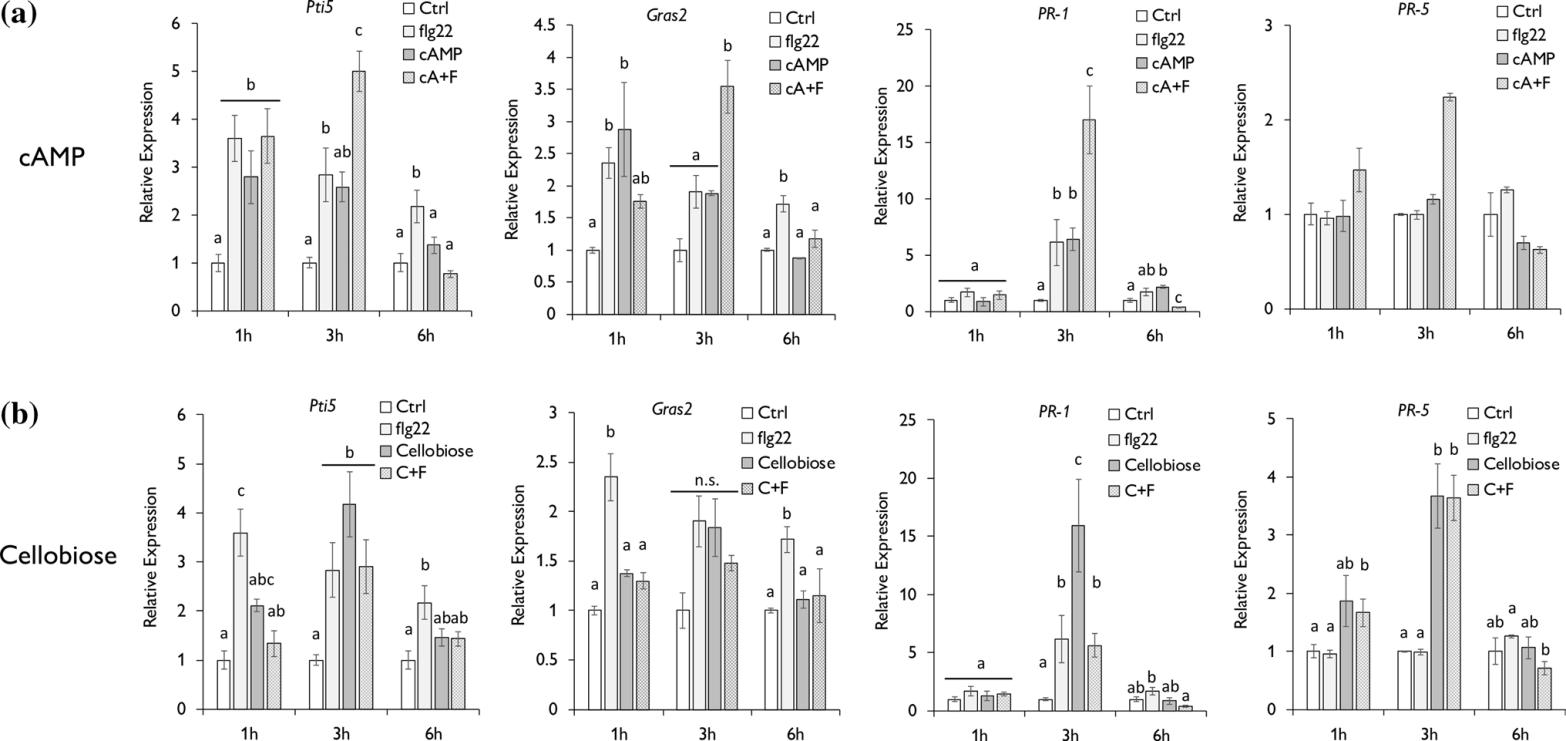
cAMP and flg22 simultaneous application induces *Pti5*, *Gras2, PR-1* and *PR-5* expression 3hpt. Gene expression study in cAMP-treated (**a**) and cellobiose-treated (**b**) plants was determined by RT-q-PCR 1, 3 and 6 h after treatment. Water (Ctrl), the 22 amino acid flagellin peptide (flg22), cAMP and cellobiose treatments were applied individually. A mixture of flg22 and cAMP (cA + F) or flagelling and cellobiose (C + F) was prepared for simultaneous application. Expression values were normalized using the housekeeping gene elongation factor 1-α (EF-1α). Error bars represent SD; letters indicate statistical differences within the treatments (ANOVA, LSD *P* < 0.01; *n* = 3)

We, therefore, hypothesized that DAMPs and PAMP co-applications would enhance the expression of PTI marker genes in tomato leaves compared to individual applications. Hence, we quantified the expression of *Pti5*, *Gras2* and the *Pathogenesis-Related protein 1* (*PR*-1) and *5* (*PR*-5)*,* PTI marker genes induced during *Pst* infection in tomato (Nguyen et al. 2010; Bektas 2021), 1, 3 and 6 h after the application of the elicitors. Consistent with this hypothesis, we observed that cAMP and flg22 co-application significantly induced the expression of *Pti5, Gras2, PR-1* and *PR-5* compared to cAMP or flg22 treatments alone at 3 hpt, being *PR-1* the most significant induction with 17 × fold compared to expression in control treatments (Fig. 4a). At 1 hpt, there was a significant upregulation of *Pti5* and *Gras2* in all treated plants, but the level of induction did not differ among the treatments (Fig. 4a). In contrast, simultaneous application of cellobiose and flg22 did not potentiate the induction generated by flg22 or cellobiose alone (Fig. 4b). Furthermore, flg22 treatments significantly induce the expression of *Pti5* 1,3 and 6 hpt, whereas cellobiose did it to *Pti5, PR-1 and PR-5* at 3 hpt. Thus, the co-application of both triggered comparable expression levels to a single application. For *Gras2*, only flg22 enhanced the expression levels 1 and 6 hpt indicating that amplification of downstream PTI responses may differ depending on the DAMP origin. Together, these data support the hypothesis that the complex mixture of a PAMP and a DAMP can amplify PTI responses in tomato plants.

### The biosynthesis of phytocytokines is potentiated by DAMPs and flg22 co-application

**Fig. 5:**
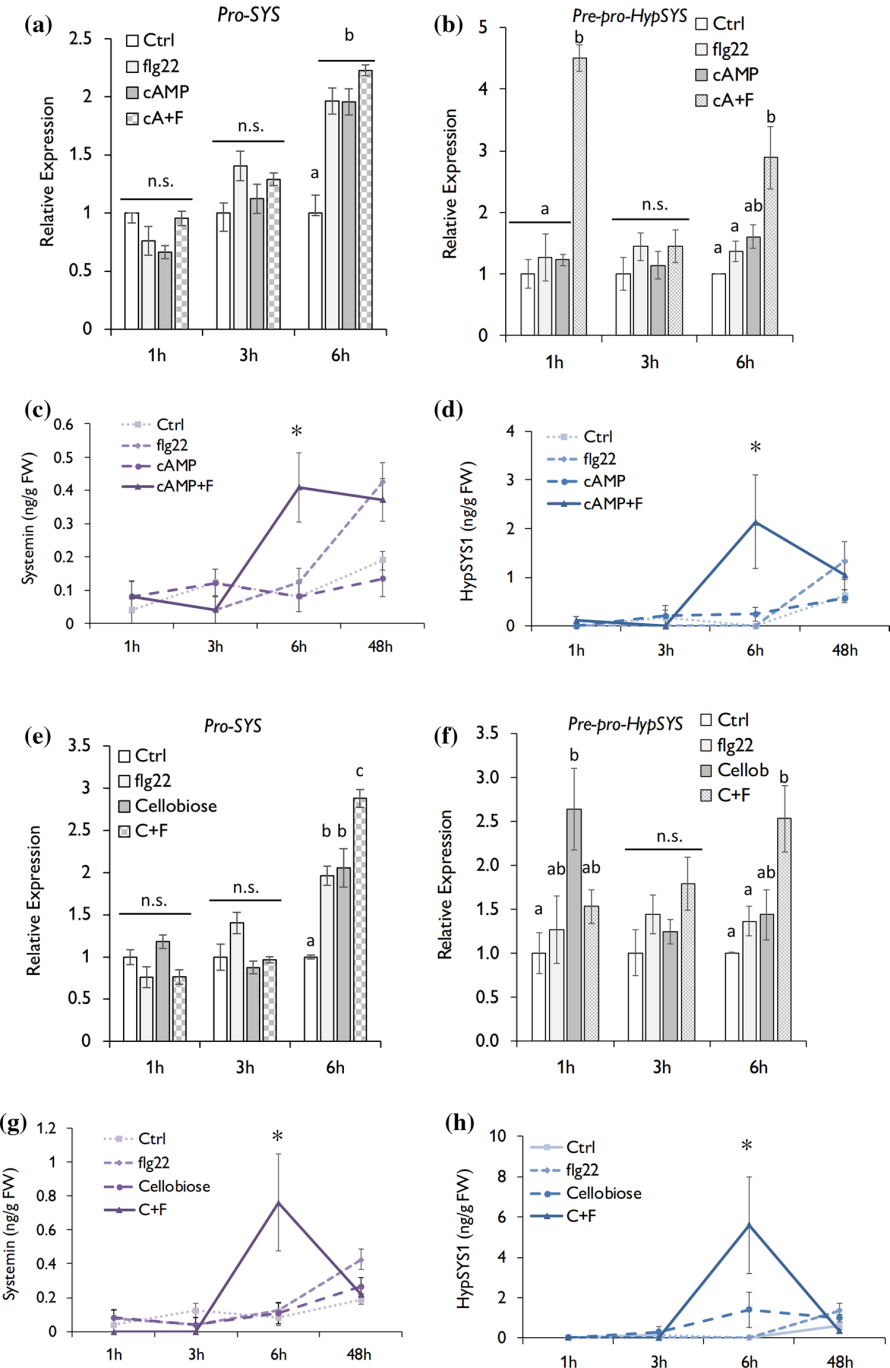
DAMPs and flg22 simultaneous application triggers the biosynthesis of secondary DAMPs. **a**–**d** Effect of cAMP and the 22 amino acid flagellin peptide (flg22) co-application, and, **e**–**h** effect of cellobiose and the 22 amino acid flagellin peptide (flg22) co-application on the gene expression of prosystemin (Pro-SYS) and hydroxyproline-rich peptide (HypSYS) and the biosynthesis of systemins and HypSYS1. **a, b, e, f** RT-q-PCR gene expression, and **c, d, g, h** the quantification of peptides levels were determined 1, 3, 6 and 24 h post treatment. Expression values for RT-q-PCR data were normalized using the housekeeping gene elongation factor 1-α (EF-1α). Bars represent means ± SD. Letters indicates statistical differences within the treatments (ANOVA, LSD *P* < 0.01; *n* = 6). Asterisks indicates statistical differences within the treatments (Student's t-test *P* < 0.01; *n* = 6)

In an effort to understand how the simultaneous perception of DAMPs and PAMPs can affect other defence-related strategies in tomato plants, we determined the expression levels of prosystemin (Pro-SYS) and hydroxyproline-rich peptide (Pre-Pro-HypSYS), precursor proteins of SYS and HypSYS, respectively (Gust et al. 2017; Zhang and Lin 2020), and quantified the levels of SYS and HypSYS1. For cAMP treatments, *Pro-SYS* expression was upregulated 6 h after independent or simultaneous application of flg22 or cAMP but with no significant differences among them (Fig. 5a). However, a significant upregulation of *Pre-Pro-HypSYS* was appreciable 1 h and 6 h after co-application of flg22 and cAMP compared to control and single applications (Fig. 5b). Similarly, the quantification of SYS and HypSYS1 showed a higher accumulation of both peptides in response to simultaneous application of cAMP + flg22 compared to individual treatments at 6 hpt (Figs. 5c, d). On the other hand, cellobiose and flg22 simultaneous application significantly induced *Pro-SYS* 6 hpt compared to the induction triggered by a single application, whereas at earlier timepoints, the levels remained unaltered (Fig. 5e). For *Pre-Pro-HypSYS*, cellobiose alone induced the expression levels 1 hpt, whereas the visible induction triggered by cellobiose and flg22 co-application 6 hpt did not significantly differ from the expression in cellobiose treated plants (Fig. 5f). Consistent with this observation, the levels of SYS and HypSYS1 were more accumulated in simultaneously treated plants than in plant with single applications at 6 hpt (Fig. 5g, h).

### DAMPs and flagellin co-application modify plant metabolic responses depending on the DAMP origin

**Fig. 6:**
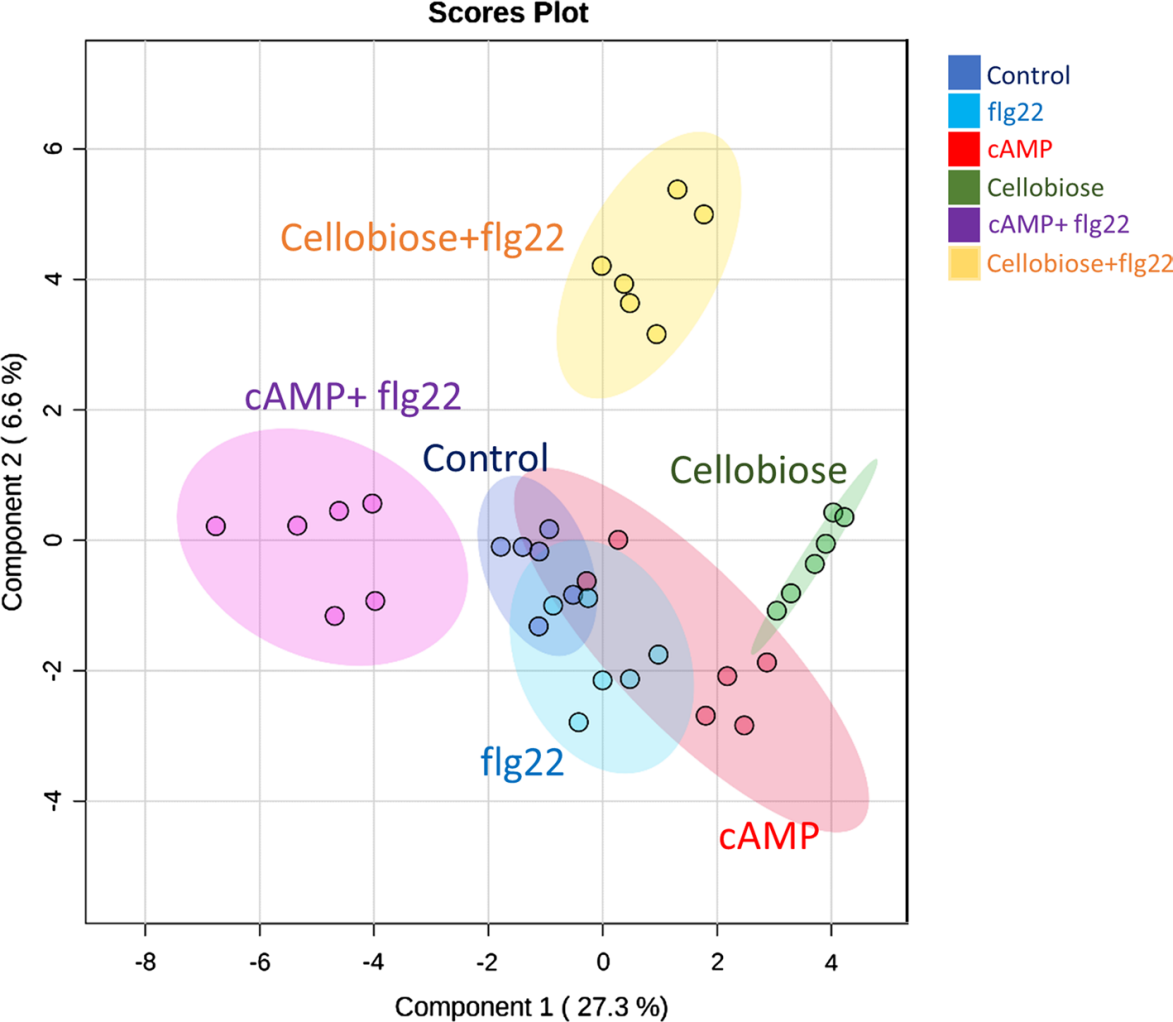
Effect of DAMPs and PAMP application on tomato plant metabolome. sPLS-DA representing ESI + and ESI- signals from untargeted metabolomics by UPLC MS/MS. Plants were treated individually with water (control), the 22 amino acid flagellin peptide (flg22), cAMP and cellobiose. A mixture solution of cAMP and flg22 (cAMP + flg22) or cellobiose and flg22 (cellobiose + flg22) was prepared for co-applications. Leaves samples were harvested 48 hpt and data points represent six biological replicates per treatment

**Fig. 7:**
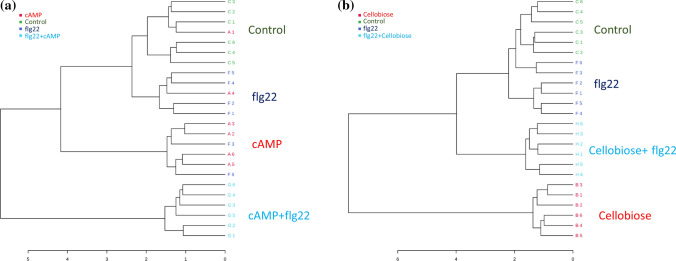
Flagellin co-application with cAMP has a more significant impact on tomato metabolome than with cellobiose. Dendrogram diagram showing hierarchical distances between **a** water (control), the 22 amino acid flagellin peptide (flg22), cAMP and a mixture solution of flg22 and cAMP (cAMP + flg22), and **b** water (control), the 22 amino acid flagellin peptide (flg22), cellobiose and a mixture solution of flg22 and cellobiose (Cellobiose + flg22). The diagram represents ESI + and ESI- signals from untargeted metabolomics by UPLC MS/MS. Leaves samples were harvested 48 h post-treatment

To further investigate the synergistic effect of DAMPs and PAMPs co-application, we performed a non-targeted metabolomic analysis 48 h after simultaneous application of cellobiose + flagellin or cAMP + flagellin. First, we determined the major sources of variations representing a sparse least square discriminant analysis (sPLS-DA), showing that the metabolic responses in plants treated with flg22 or cAMP did not cluster separately from water-treated plants (Fig. 6). In contrast, plants treated with cellobiose did show a strong metabolic rearrangement compared to control plants (Fig. 6). Furthermore, plotting the two principal components of the data is enough to separate cAMP + flg22 and cellobiose + flg22 simultaneous treatments in different clusters compared to control plants indicating that cAMP and cellobiose might amplify flg22-dependent metabolic responses, although it is unclear whether cellobiose by itself has the most substantial effect (Fig. 6). To answer this question, we performed a dendrogram clustering to show a hierarchical relationship between treatments. The diagram confirmed that the simultaneous application of cAMP + flg22 amplifies single flg22 or cAMP responses (Fig. 7a). However, it also showed that cellobiose treatments trigger more significant metabolic changes than those triggered by the co-application with flg22 (Fig. 7b). A detailed analysis of the untargeted metabolomics revealed that co-applications cAMP + flg22 and cellobiose + flg22 significantly impacted the phenylpropanoid biosynthetic pathway, containing numerous compounds with significantly different accumulations, such as chlorogenic acid, cinnamic acid, coniferyl alcohol and salicylic acid (Tables 1 and 2) revealing a metabolomic rearrangement after the simultaneous perception of DAMPs and PAMPs.

**Table 1:** Identified phenylpropanoids in non-targeted metabolomic analysis. cAMP and flagellin treatment

Phenylpropanoids	cAMP	Flagellin	F + A
Ferulic acid	0.9	0.9	0.8
Chlorogenic acid	0.6^a^	0.9	1.3^b^
Caffeic acid	0.6^a^	0.7^a^	0.8^a^
Cinamic acid	1.3	1.2	1.5
Conyferil alcohol	1.2	1.2	1.5^b^
4-Coumaroil alcohol	1.0	0.9	0.9
Salicylic acid	1.0	1.2	2.3^b^

^a^Significantly different compared to control; Student's *t* test *P* < 0.01; *n* = 6

^b^Significantly different compared to cAMP and Flagellin; Student's *t* test *P* < 0.01; *n* = 6

**Table 2:** Identified phenylpropanoids in non-targeted metabolomic analysis. Cellobiose and flagellin treatment

Phenylpropanoids	Cellobiose	Flagellin	F + C
Ferulic acid	0.8^a^	0.9	0.8^a^
Chlorogenic acid	0.2^a^	0.9	0.9^b^
Caffeic acid	0.5^a^	0.7^a^	0.7^a^
Cinamic acid	1.1	1.2	2.4^b^
Conyferil alcohol	1.0	1.2	0.8^a^
4-Coumaroil alcohol	0.9	0.9	0.7
Salicylic acid	1.1	1.2	0.8

^a^Significantly different compared to control; Student's *t* test *P* < 0.01; *n* = 6

^b^Significantly different compared to cellobiose and Flagellin; Student's *t* test *P* < 0.01; *n* = 6

### Cellobiose and cAMP treatments induces resistance against *P. syringae* pv. *tomato* DC3000

**Fig. 8:**
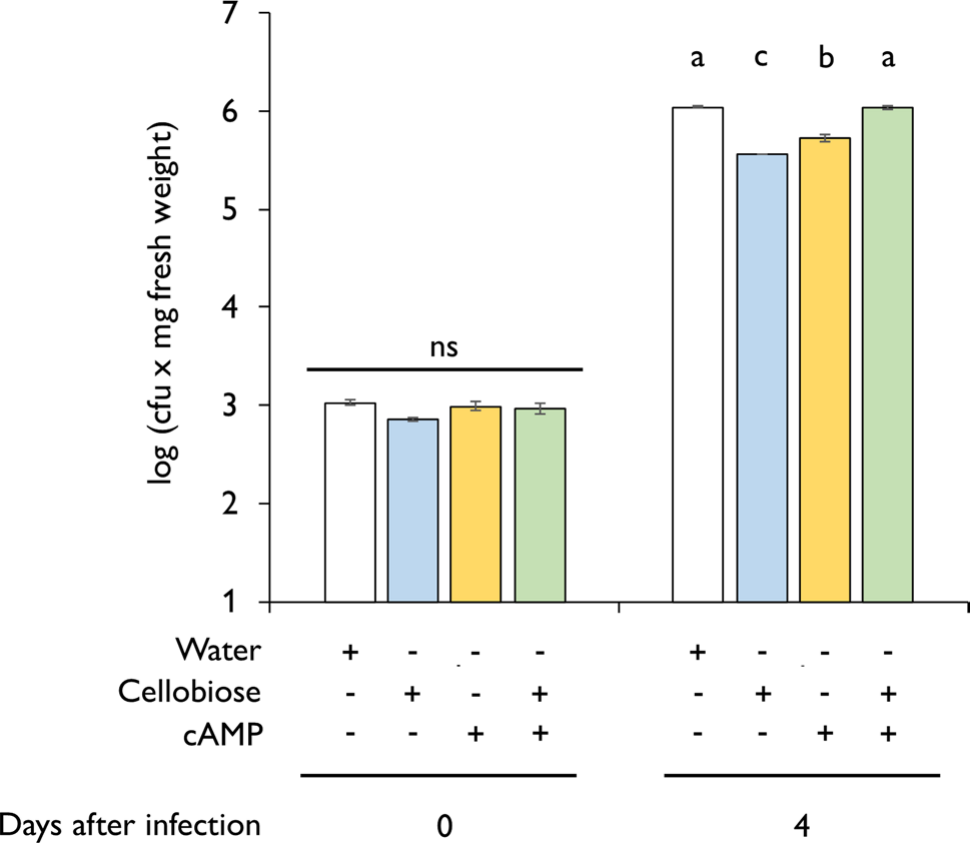
Cellobiose and cAMP induces tomato resistance against *P. syringae* pv*. tomato* DC3000. Four-week-old tomato plants were infected in the third and fourth true leaves with the hemibiotrophic bacteria *Pst*. The number of cfu were determined 0 and 4 days after infection. Water, cAMP and cellobiose treatments were applied 24 h before the infection. Bars represent means ± SD. Letters indicates statistical differences within the treatments (ANOVA, LSD *P* < 0.01; *n* = 6)

Finally, we tested the biological relevance of DAMPs-IR in tomato against the hemibiotrophic bacteria *P. syringae* pv. *tomato* DC3000. After a cell disruption upon pathogen attack, the intercellular content and cell wall derived products are released to the apoplast and perceived by adjacent-intact cells, triggering an alarm state of the plant. Our goal was to determine whether the DAMPs perception in intact cells induced resistance against *Pst*. Hence, we treated tomato plants with either 100 µM cellobiose, 25 µM cAMP or a mixture solution of both DAMPs 24 h before the inoculation with 1 × 10^5^ cfu of *Pst*. Four days after the infection, we macerated the infected leaves in MgSO_4_ and plated the leaf extract in solid media plates. The colony counting revealed that individual treatments with cellobiose and cAMP reduced bacterial growth compared to control/water-treated plants (Fig. 8). Among both DAMPs, cellobiose significantly reduced *Pst* growth compared to cAMP treated plants. We also tested whether the co-application of both DAMPs, cAMP and cellobiose, could induce resistance against *Pst*, but contrary to what we expected the mixture solution of DAMPs did not reduce the number of *Pst* colonies, showing no synergy after the simultaneous perception of both endogenous signals (Fig. 8).

## Discussion

This study aimed to test the hypothesis that self-damage perception amplifies PTI responses after simultaneous perception of DAMPS and PAMPs. Here, our data show that DAMPs recognition strengthens flagellin-derived PTI responses and stimulates the biosynthesis of systemin and HypSYS1 in tomato. Additionally, the non-targeted metabolomic analysis unveils specificity in the generation of defence-related metabolites, depending on the origin of the DAMP. Thus, based on our results, we can conclude that plants benefit from the simultaneous perception of both signals boosting primary and secondary inducible defences. However, the specificity shown at the gene expression and the metabolic level indicates that individual studies should be performed to understand the complete molecular and metabolic response after self-damage perception.

Here, we have demonstrated that the co-application of PAMPs and DAMPs significantly amplifies the generation of apoplastic H_2_O_2_ and MAPK phosphorylation. In tomato, there are eight *Rbohs* identified, but with different expression pattern depending on the challenge. The expression of *RbohA*, B and C are induced after *B. cinerea* and *Pst* infection. However, VIGS (virus-induced gene silencing)-based silencing of *RbohB* resulted in reduced resistance to *B. cinerea,* but silencing of other *Rbohs* did not affect the resistance (Li et al. 2015). It is important to note that not many research studies have investigated the consequences of simultaneous DAMPs and PAMPs perception. For example, Azevedo Souza et al. (2017) showed that co-treatments of cellobiose with two different PAMPs, flagellin or chitooligomers, increase Ca^2+^ influx compared to individual treatments. Similarly, the co-existence of a phytocytokine, OsPEP3, and *Mythimna loreyi* oral secretion potentiates MAPK phosphorylation and increases JA-Ile levels in rice cells (Shinya et al. 2018). One additional study in tomato plants showed that the simultaneous application of pectin-derived oligogalacturonides and chitooligosaccharides induces the accumulation of defence-related proteins and the expression levels of salicylic acid (SA)-related genes, among other PR genes (Van Aubel et al. 2016). Although we cannot exclude the involvement of principal defence-related hormones in our system, further studies in tomato plants should be conducted to confirm which hormonal-related pathways are involved. Additionally, we have also shown that simultaneous application of DAMPS and flagellin increases the expression of *Gras2* and *Pti5*, *PR-1* and *PR-5* (Nguyen et al. 2010; Bektas 2021). A possible explanation for the amplification of PTI responses is that DAMPs recognition triggers the accumulation of extracellular PAMP receptors, boosting PTI responses. Another plausible explanation is that DAMPs and PAMPs simultaneous perception generates more powerful responses because the plant's response to DAMPs benefits from the PTI pathway activated by PAMPs. A recent publication has demonstrated that the perception of flg22 MAMP in Arabidopsis roots increased the number of extracellular receptors in adjacent cells (Zhou et al. 2020). Although it should be confirmed, the conclusions obtained from this manuscript, together with our results, made us speculate that self-damage recognition may turn the plant more sensitive to external threats.

When analysing the plant metabolomic responses after the co-application of flagellin and cAMP or cellobiose, we observed that cAMP amplifies flg22 metabolic responses revealing that the simultaneous perception of self- and non-self-damage boosts downstream PTI responses depending on the origin of the DAMP. This metabolomic behaviour could be associated with the idea that plants only generate downstream defence responses in case of correlation between signals, following an energy-saving strategy. The simultaneous recognition of one specific DAMP and one specific PAMP could give the plant valuable information about endogenous damage and the attacker triggering specific defensive responses against one particular pathogen. On the other side, the perception of one specific DAMP not correlated with a PAMP may result in a weaker defence response as part of the energy-saving strategy. In this case, the simultaneous perception of cellobiose and flg22 may not be correlated, and plants do not trigger a significant metabolic response. Based on other studies, flagellin treatments induce the phenylpropanoids biosynthesis pathways and decrease the content of most amino acids (Misra et al. 2016). Here, we were not able to detect amino acids, but we observed that several phenylpropanoids are differentially accumulated in flg22 + cellobiose and flg22 + cAMP compared to flagellin-treated plants (Tables 1 and 2). Although we need a deeper study to totally understand the impact in the plant metabolome, we hypothesized that the perception is specific on the origin of the DAMP.

We hypothesize that co-applications of DAMPs and PAMPs can strengthen downstream PTI responses. Consistent with this hypothesis, the data supports that the production of inducible phytocytokines is potentiated by the simultaneous perception of self- and non-self-molecular patterns. Here, we found that co-applications with cellobiose enhanced the flagellin-dependent *Pro-SYS* induction and accumulated higher levels of SYS. Prosystemin is a gene encoding a protein precursor of the 18 amino acid polypeptide SYS (McGurl and Ryan 1992). SYS was discovered 30 years ago as an inducer of proteinase inhibitors, and it has been demonstrated that it coordinates local and systemic immune defences (McGurl et al. 1992; Zhang and Lin 2020). However, systemin's role in modulating defence responses against bacteria and other pathogens is still unexplored. Recent reports showed that SYS treatments trigger SA, which play a critical role in defence signalling against biotrophic pathogens, and induce resistance against several necrotrophic pathogens (Pastor et al. 2018; Coppola et al. 2019; Pastor-Fernández et al. 2020). Systemin is well known to induce systemic JA-dependent responses after herbivory or necrotrophic attack but, to confirm its role as an elicitor of SA signalling, future experiments should provide more evidence. We further found that simultaneous flg22 application with both DAMPs enhanced the expression levels of *Pre-Pro-HypSYS* and triggered the accumulation of HypSYS1 compared with individual treatments. Pre-Pro-HypSYS is another inducible phytocytokine that is finally processed in three different peptides in tomato (Narváez-Vásquez et al. 2005). The HypSYS peptides induce the synthesis of proteinase inhibitor proteins and other defence-related responses against insects (Narváez-Vásquez et al. 2007; Pearce et al. 2007). Interestingly, treatments with synthetic HypSYS activate insect-related responses and pathogen-inducible defences such as the enzymatic activity of PAL and the gene expression of PAD4 and NPR1 (Bhattacharya et al. 2013). Therefore, despite the signalling role that these inducible peptides have against herbivorous insects, these results indicate that these phytocytokines may also have a signalling function against pathogenic microbes.

Here, we have demonstrated that cellobiose and cAMP act as DAMPs signals separately in tomato, and individual applications of both induce resistance in tomato plants against the hemibiotrophic bacteria *Pseudomonas syringae* pv*. tomato* DC3000. DAMPs are released from attacked cells to the apoplast and, their perception by adjacent intact cells induces an alarm state in the plant (Gust et al. 2017). Previous studies demonstrated that intracellular cAMP acts upstream SA (Mauch-Mani and Slusarenko 1996; Jiang et al. 2005; Huang et al. 2010; Shine et al. 2016). Increasing levels of cAMP trigger SA accumulation in response to Verticillium toxins, and plants with low cAMP strongly reduce SA concentration and PR-1 expression in response to different pathogens ( Sabetta et al. 2016). Additionally, cAMP promotes CNGC Ca^2+^ current, leading to cytosolic Ca^2+^ elevation and NO generation (Ma et al. 2009). It has been proposed that the critical CNGC-mediated Ca^2+^ conductance can also occur through PAMP perception, explaining the induction of defensive responses and the increased resistance of tomato against *Pst.* On the other hand, Azevedo Souza et al. (2017) demonstrated that cellobiose treatments induce resistance against *Pst* in 2-week-old Arabidopsis seedlings. These findings demonstrate that using DAMPs as an environmentally friendly biostimulants is possible. However, contrary to what we expected, the combination of both treatments did not have a synergistic effect and restored a control phenotype. A possible explanation is that cAMP and cellobiose are DAMPs from different origins, and the combination of both could mislead the target for the plant defences. It is also possible that a negative interaction between both signals leads to an antagonistic effect against hemibiotrophic pathogens. Thus, further work is needed to explore the potential application of DAMPs combinations as a pathogen or insect biostimulants.

In summary, these results revealed that a mixture solution of self- and non-self-danger molecules strengthens plant innate immunity responses. This is of particular interest since very few studies have shown evidences to explain why plants can perceive DAMPs and PAMPs to activate similar defence responses. Our data fit with one of the four hypotheses proposed by Duran-Flores and Heil (2016) to give an explanation for such fascinating phenomenon. Additional studies testing other DAMPs and PAMPs will be necessary to assess whether this phenomenon depends on the combination of the DAMP and PAMP or it is conserved among different molecular patterns. The recognition of damaged self- and non-self-molecular patterns is a general strategy for the activation of the plant innate immunity, and our study demonstrates that the simultaneous perception of both is a key part to potentiate it.

### Author contribution statement

VP, RC and JG performed the experiments. VP and JG wrote the manuscript. JG designed the experiments.

## Supplementary Information

Below is the link to the electronic supplementary material.

Supplementary file1 (TIF 685 KB)

Supplementary file2 (TIF 848 KB)

Supplementary file3 (TIF 672 KB)

## Data Availability

The datasets generated during and/or analysed during the current study are available from the corresponding author on reasonable request.

## Abbreviations

DAMPs: Damage-Associated Molecular Patterns
DTI: DAMP-Triggered Immunity
hpt: Hours post-treatment
PAMPs: Pathogen-Associated Molecular Patterns
PTI: PAMP-Triggered Immunity
Pti5: Pto Interacting protein 5
Pre-Pro-HypSYS: Hydroxyproline-rich peptide
Rboh’s: Respiratory Burst Oxidase Homologs
SYS: SYSTEMIN
