# Supplementary figures and images for: The simultaneous perception of self- and non-self-danger signals potentiates plant innate immunity responses
Source: Planta. 2022 Jun 13;256(1):10. doi: 10.1007/s00425-022-03918-y (PMC9192368; doi:10.1007/s00425-022-03918-y)

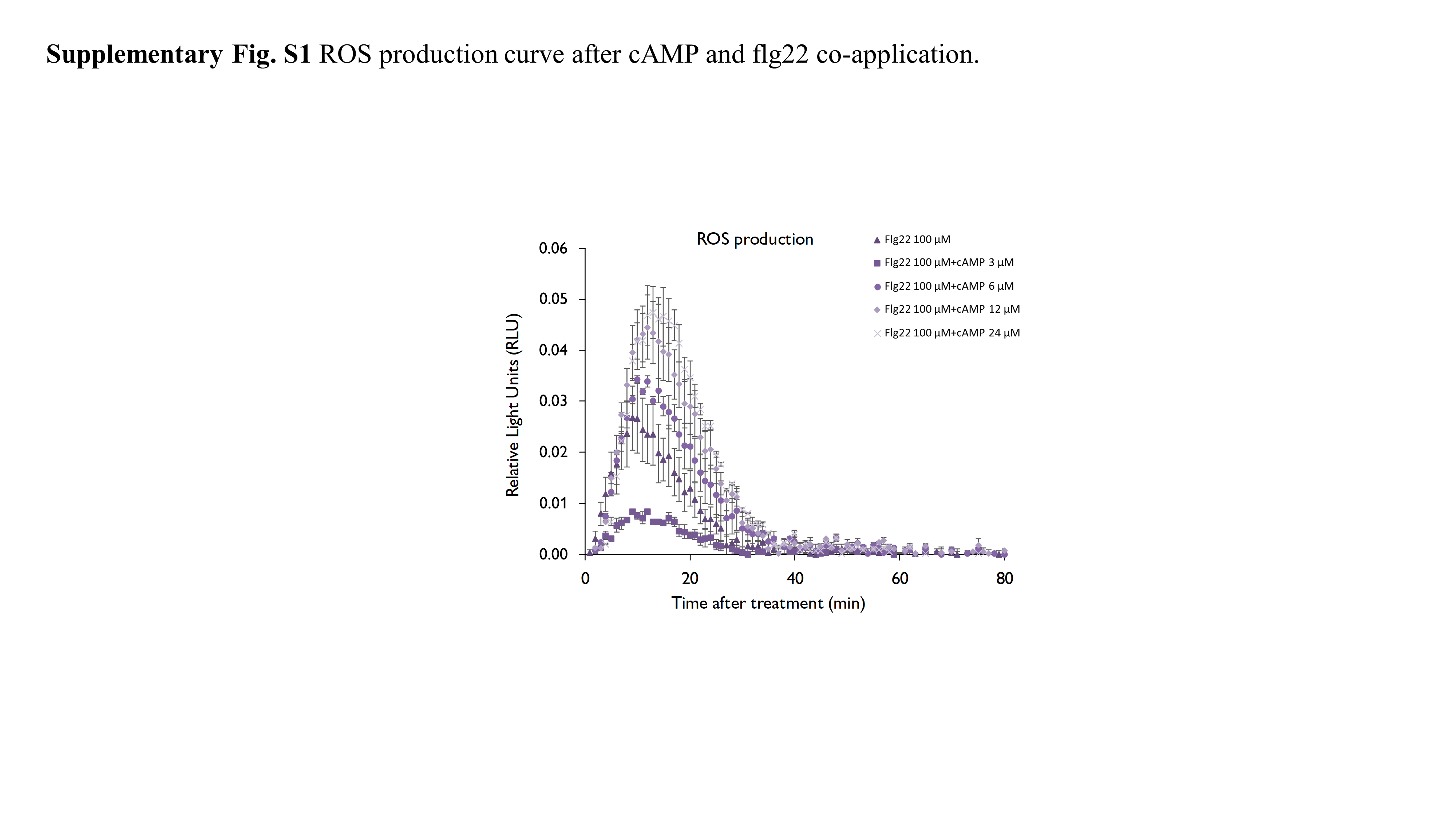

Supplement: Supplementary file 1 — Supplementary file1 (TIF 685 KB) [file 425_2022_3918_MOESM1_ESM.tif]

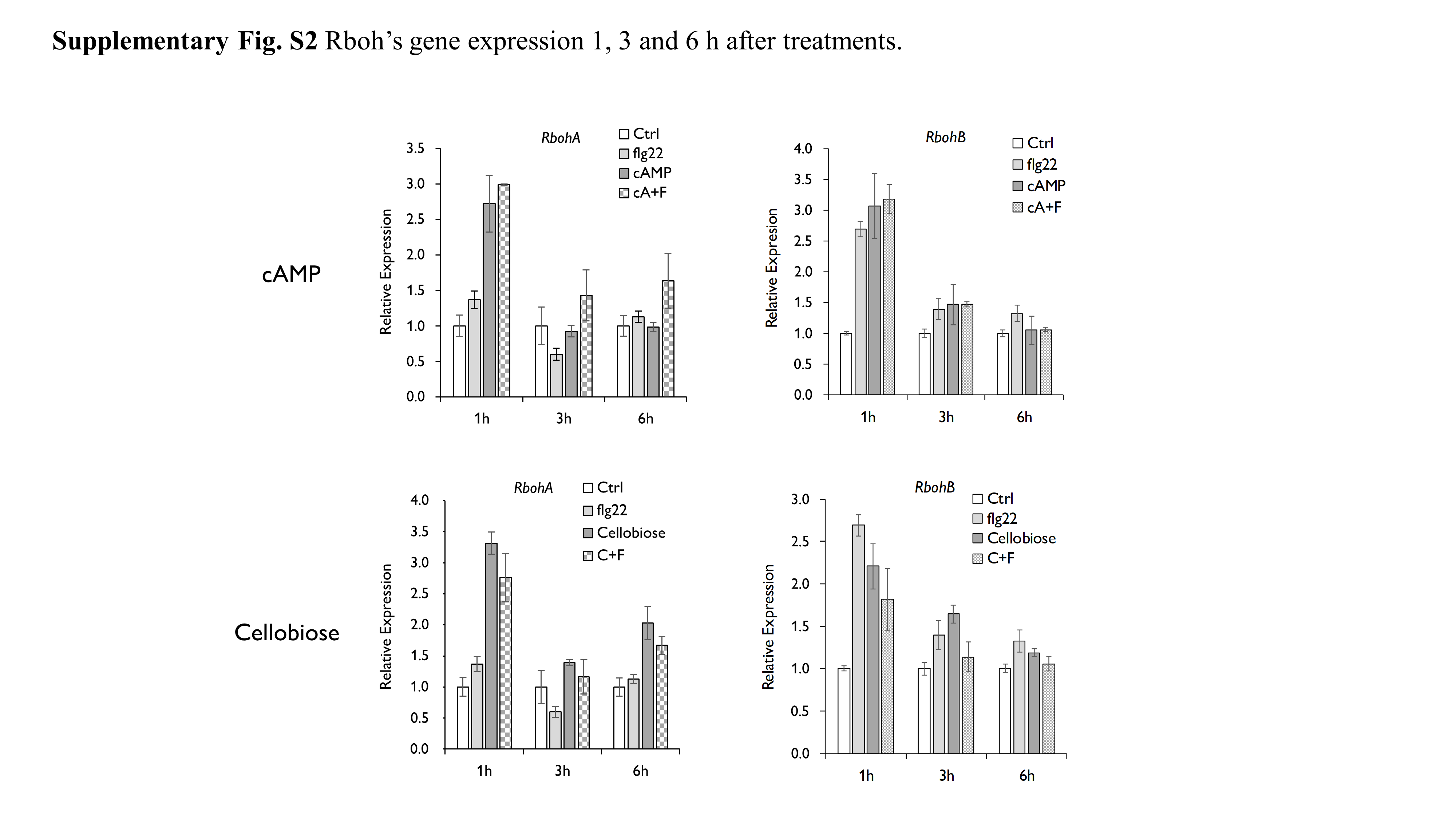

Supplement: Supplementary file 2 — Supplementary file2 (TIF 848 KB) [file 425_2022_3918_MOESM2_ESM.tif]

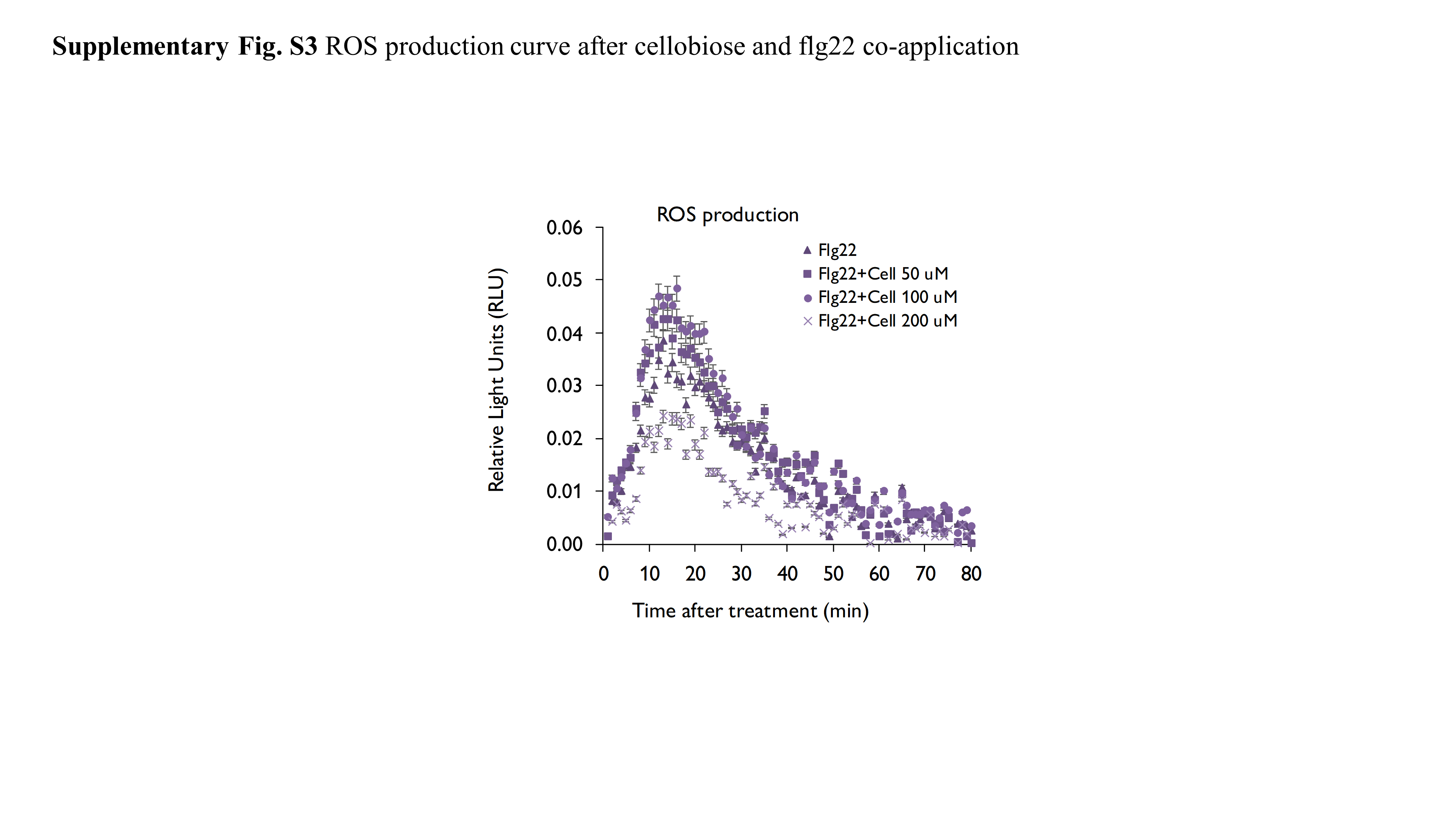

Supplement: Supplementary file 3 — Supplementary file3 (TIF 672 KB) [file 425_2022_3918_MOESM3_ESM.tif]
